# Burden of Influenza and Respiratory Syncytial Virus Infection in Pregnant Women and Infants Under 6 Months in Mongolia: A Prospective Cohort Study

**DOI:** 10.1371/journal.pone.0148421

**Published:** 2016-02-05

**Authors:** Liling Chaw, Taro Kamigaki, Alexanderyn Burmaa, Chuluunbatiin Urtnasan, Ishiin Od, Gunregjaviin Nyamaa, Pagbajabyn Nymadawa, Hitoshi Oshitani

**Affiliations:** 1 Department of Virology, Tohoku University Graduate School of Medicine, Sendai, Japan; 2 National Influenza Center, National Center of Communicable Diseases, Ulaanbaatar, Mongolia; 3 Baganuur District, Ulaanbaatar, Mongolia; 4 Mongolian Academy of Medical Sciences, Ulaanbaatar, Mongolia; University of Hong Kong, HONG KONG

## Abstract

**Background:**

Pregnant women and infants under 6 months are at risk of influenza-related complications. Limited information exists on their community burden of respiratory viruses.

**Methods and Findings:**

This prospective, observational open cohort study was conducted in Baganuur district, Ulaanbaatar, Mongolia during 2013/14 and 2014/15 influenza seasons. Influenza-like illness (ILI) and severe acute respiratory infection (sARI) were identified by follow-up calls twice a week. For those identified, influenza and respiratory syncytical virus (RSV) were tested by point-of-care test kits. We calculated overall and stratified (by trimester or age group) incidence rates (IR) and used Cox proportional hazard regression for risk factor analyses. Among 1260 unvaccinated pregnant women enrolled, overall IRs for ILI, sARI and influenza A were 11.8 (95% confidence interval (C.I):11.2–12.4), 0.1 (95%C.I:0.0–0.4), and 1.7 (95%C.I:1.5–1.9) per 1,000person-days, respectively. One sARI case was influenza A positive. IRs and adjusted hazard ratios (Adj.HR) for ILI and influenza A were lowest in the third trimester. Those with co-morbidity were 1.4 times more likely to develop ILI [Adj.HR:1.4 (95%C.I:1.1–1.9)]. Among 1304 infants enrolled, overall ILI and sARI IRs were 15.2 (95%C.I:14.5–15.8) and 20.5 (95%C.I:19.7–21.3) per 1,000person-days, respectively. From the tested ILI (77.6%) and sARI (30.6%) cases, the overall positivity rates were 6.3% (influenza A), 1.1% (influenza B) and 9.3% (RSV). Positivity rates of influenza A and RSV tend to increase with age. sARI cases were 1.4 times more likely to be male [Adj.HR:1.4 (95%C.I:1.1–1.8)]. Among all influenza A and RSV positive infants, 11.8% and 68.0% were respectively identified among sARI hospitalized cases.

**Conclusion:**

We observed low overall influenza A burden in both groups, though underestimation was likely due to point-of-care tests used. For infants, RSV burden was more significant than influenza A. These findings would be useful for establishing control strategies for both viruses in Mongolia.

## Introduction

Pregnant women and infants under 6 months old are at risk of influenza-related complications, leading to hospitalizations [1–4]. This impact was observed among pregnant women during seasonal influenza season [5, 6] and more apparently during the pandemic (H1N1) 2009 [7]. Briefly, pregnant women in the third trimester were found to be 3–4 times more likely to be hospitalized for an acute cardiopulmonary illness during the inter-pandemic influenza season, when compared to post-partum women [6]. Also, pregnant women with influenza A(H1N1)pdm09 infection were about seven times more likely to be hospitalized and two times more likely to die, when compared to non-pregnant women of reproductive age [8].

Also, infants under 6 months are known to have higher hospitalization rates for both seasonal influenza and respiratory syncytical virus (RSV). In the United States, the influenza-related hospitalization rate was estimated to be 4.5 per 1,000 children under 6 months old [9], while that due to RSV was 16.9 per 1,000 children [10]. In a birth cohort in Kenya, infants under 6 months had the highest rate of RSV-associated disease [11].

Maternal influenza vaccination is one of the safe and cost-effective interventions of protecting both pregnant women as well as infants under 6 months, to whom direct influenza vaccination is not indicated [4, 12]. It was shown to reduce the frequency of febrile respiratory illnesses in pregnant women [13], as well as the frequency of influenza infection [13, 14] and hospitalization [15, 16] in infants under 6 months. Recognising its two-fold benefit, the WHO Strategic Advisory Group of Experts (SAGE) on immunization recommended that pregnant women should be vaccinated against influenza at any pregnancy stage [17]. Despite this, vaccination rate is still low in both developed [18] and developing [3] countries. Maternal RSV vaccination, when it is available, is also one useful strategy to protect infants under 6 months [19, 20]. RSV subunit vaccines are currently being developed for the adult population, including pregnant women [21].

There are a few reasons why Mongolia is chosen as the study site. First, maternal and child health is currently one of the important health issues in Mongolia due to its high birth rate and population growth. Between 2005 and 2014, the number of live births increased at an average rate of 6.4% [22, 23]. In 2014, the estimated annual birth rate is 20.9 per 1,000 persons [24]. Second, it was previously shown that children < 5 years old in Mongolia have the highest incidence of influenza-associated illness [25]. Third, the burden among pregnant women has never been assessed in the country, even though 24.1% of all influenza A(H1N1)pdm09-confirmed deaths in Mongolia were pregnant [26]. During the study period, the country’s national influenza vaccination policy did not include pregnant women as one of the recommended vaccine recipients [27].

Limited data exists on the disease burden of respiratory viruses for both populations at the community level. Therefore, we conducted a prospective observational cohort study on acute respiratory viruses for both groups during the 2013–14 and 2014–15 influenza seasons in a Mongolian community. The study objectives were to determine the incidence and risk factors of influenza-like illness (ILI), severe acute respiratory infection (sARI) and virus-positive cases (influenza A, influenza B and RSV) in pregnant women and infants under 6 months.

## Material and Methods

### Study site

Baganuur is a semi-urban district located at about 130 km away from the central part of Ulaanbaatar, Mongolia’s capital city. In 2013, it had an estimated population of 27,440. Medical services are provided mainly by one district hospital and four primary healthcare centers called Family General Practitioners (FGPs). We conducted the study in this district for two consecutive influenza seasons (October 1st 2013—April 30th 2014 and October 1st 2014—April 30th 2015).

### Data collection

In Mongolia, all pregnant women and children < 2 years old are required to register and undergo medical check-ups at their FGPs, which are assigned based on their official residential address. All four FGPs in the district were used as the study entry point. All residents who registered at the FGPs and met the inclusion criteria (being pregnant or aged < 24 weeks during the study period) were enrolled. To include those who had already registered and those newly registered during the study period, enrolment was carried out continuously throughout the study period. Follow-up ends if any of the following occurred: a woman was no longer pregnant, a child reached 24 weeks of age, or any participant moved away from Baganuur district.

At the time of enrolment, a baseline questionnaire was administered to all participants to collect their demographic information. Prenatal information and household member demographics were also collected from pregnant women for both seasons. For infants under 6 months, information on birthweight, gestational age at delivery and presence of birth defects were collected for both seasons, while the household member demographics were collected only during the 2014/15 season. Household members who were < 18 years old were categorized as: young child (until 2 years), kindergarten-age (2–5 years), and school-age (6–17 years).

For pregnant women, initial antenatal examination is carried out by an obstetrician in the district hospital on their first check-up, and the results are recorded in the antenatal check-up record book. This book served as the major source of baseline data in this study. The obstetrician classifies all pregnant women as either normal or high risk to indicate whether she would undergo routine or extensive antenatal check-ups, respectively. The first day of the last menstruation was used to determine the start date of pregnancy and the trimester stages were defined as: first (0–13 weeks), second (14–26 weeks), and third (27 weeks and above). Baseline height and weight information were also collected to calculate the body mass index (BMI). We defined pregnant women as with co-morbidity if she has any of the following medical conditions that pre-disposes them to influenza-related complications [28]: pulmonary, cardiovascular, renal, and metabolic conditions.

For infants under 6 months, low birthweight was defined as those born weighing < 2500g. Three categories were defined for term of pregnancy [29, 30]: preterm, early term, and full term.

The primary outcome in this study was the occurrence of a case positive for either influenza A, influenza B or RSV. The secondary outcome was the occurrence of an ILI or a sARI case. An ILI case was defined as the sudden onset of fever of ≥ 38°C, or with history of fever, and cough within the last 7 days. A sARI case was defined as having ILI symptoms and requires hospitalization.

Throughout the study period, scheduled follow-up calls were carried out twice a week (one call every 2–5 days) to actively identify any ILI episodes among the study participants. When an ILI episode was identified, a timely home visit was made to interview him/her with a case report form and to collect a nasopharyngeal swab for on-site testing with a point-of-care test kit (QuickNavi^™^–Flu + RSV, Denka Seiken Co. Ltd). This commercially available kit enables simultaneous detection of influenza A, influenza B and RSV. In the subsequent two follow-up calls, the resolution of ILI symptoms was also recorded. If the ILI case had recovered from symptoms on the day of follow-up call, that date was used as the date of symptom resolution.

At the district hospital, information on sARI episodes (admission and discharge dates, symptoms presented, clinical indicators, diagnosis at discharge and outcome, point-of-care test results) were collected. There were some cases who initially visited the FGP for testing, then later referred for hospitalization. An episode whose interval between FGP visit and hospital admission is < 7 days was considered as the same illness episode.

The district hospital and all four FGPs in Baganuur are also a part of the national ILI surveillance program in Mongolia. Nasopharyngeal swabs were collected randomly from ILI cases and routinely tested for influenza virus using real-time polymerase chain reaction (PCR). Some randomly selected samples were further tested for other respiratory viruses, including RSV. According to this surveillance [31], three influenza strains [A(H1N1)pdm09, A(H3N2), and B] were detected during the 2013/14 season, while A(H3N2) and RSV were detected during the 2014/15 season (S1 Fig). Some of our study participants (5.6%) were tested under this surveillance, particularly during periods of limited test kit availability. These results were also collected and combined into the analyses.

All field staff involved had been trained with participant interviewing as well as sample collecting, testing and result interpretation. The field supervisor monitored all activity from data collection to encoding.

### Ethical Approval

Ethical approval for this study was obtained from the Ethics Committee of Tohoku University, Graduate School of Medicine, Sendai, Japan (2013-1-253), the Scientific Committee of National Center of Communicable Diseases, Mongolia (14/126), and the Ministry of Health, Mongolia (2014–02). Written informed consent was obtained from eligible participants or their parents/guardians before study enrolment as well as from identified ILI and sARI cases before data and sample collection.

### Statistical analysis

Overall incidence rates (IR) were derived by dividing the actual number of cases detected with the total person-days at risk, and 95% confidence intervals (C.I) were calculated using exact methods. Total person-days were calculated based on the study entry and leaving dates. For participants with ILI or sARI, the symptomatic period was considered as days not-at-risk of infection and therefore not included in the denominator. For those with missing symptom resolution dates, we set 7 days as the duration of symptoms, based on previous studies on adults [32] and children < 1 year old [33]. For sARI cases, we defined the symptomatic period as the number of days hospitalized.

We also investigated the risk factors of getting ILI, sARI and virus positive cases. Participants who did not have ILI symptoms (non-ILI group) were initially compared univariately with the ILI cases and also with each virus positive cases (S3 and S4 Tables). sARI cases were only investigated for infants under 6 months, and they were compared univariately with those who were not hospitalized with sARI (non-sARI group) (S4 Table). Parametric (Student’s *t* and chi-square) and non-parametric (Wilcoxon rank sum and Fisher’s exact) tests were used whenever appropriate. Factors with a *p*-value of ≤ 0.1 and deemed relevant for acquiring illness episodes were then included in the Cox proportional hazards (PH) regression model. In all models runs, non-ILI or non-sARI were used as the comparison group. Participants with multiple episodes within each season were also accounted for, in both un-adjusted and adjusted models. To ensure that the model assumptions were met, diagnostic tests on the proportional hazards assumption were done and the residuals were also checked. For pregnant women, all co-variates except age at enrolment were categorically included in the final Cox PH model. Alternative model runs using categorized age quartiles were consistent with the main results reported. For infants under 6 months, all co-variates included were categorical. A *p*-value of < 0.05 was considered statistically significant.

We hypothesize that trimester stage (for pregnant women) and age group (for infants under 6 months) could contribute to differences in infection risk. Therefore, we also stratified the IR calculations accordingly and included them as the time-dependent variable in the Cox PH model. Microsoft Excel and R software, version 3.2.0 [34] were used for all analyses.

## Result

### Distribution of ILI and sARI cases, and circulating viruses in both populations

**Fig 1:**
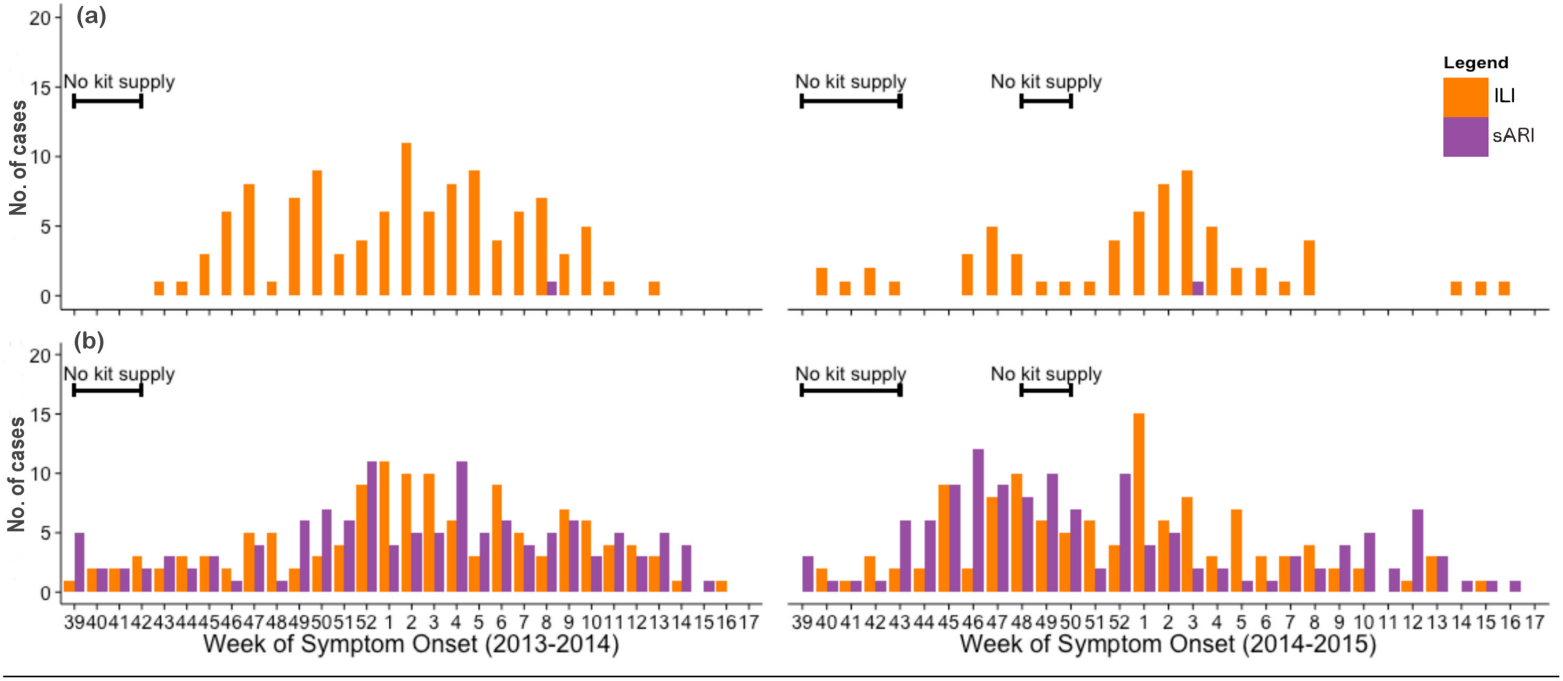
The weekly number of influenza-like illness (ILI) [orange] and severe acute respiratory infections (sARI) [purple] cases during the 2013/14 (left) and 2014/15 (right) seasons, for pregnant women (a) and infants under 6 months (b). The black lines indicate periods of limited point-of-care test kit supply, thus testing was compromised in those weeks.

Fig 1 shows the weekly number of ILI and sARI cases in pregnant women and infants under 6 months during the study period. Most ILI and sARI cases tend to occur between November to March, coinciding with winter months in Mongolia. The temporal trend of sARI correlated with that of ILI in infants (r^2^ = 0.71) while there were only 2 sARI cases observed in pregnant women.

**Fig 2:**
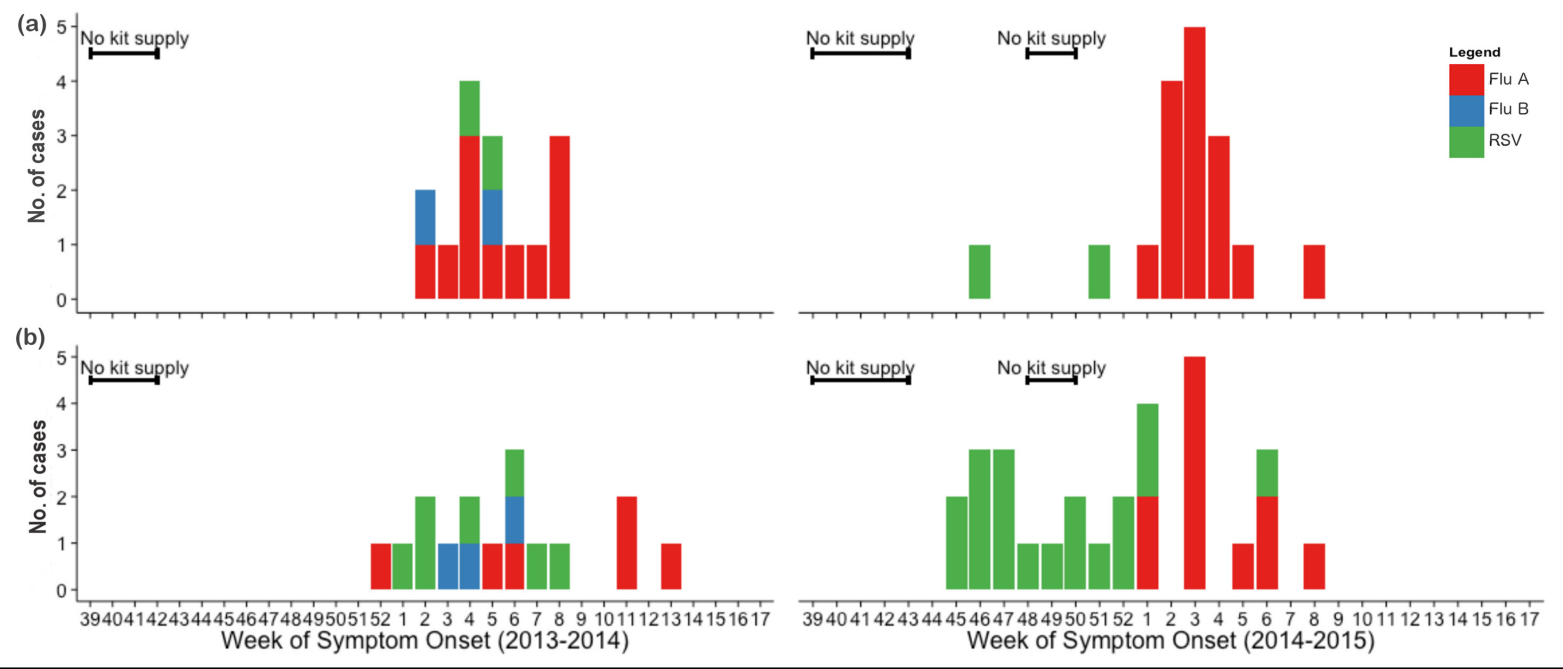
The weekly number of virus positives detected from ILI and sARI cases during the 2013/14 (left) and 2014/15 (right) seasons, for pregnant women (a) and infants under 6 months (b). The bars show the number of positive cases for influenza A (red), influenza B (blue) and RSV (green). The black lines indicate periods of limited point-of-care test kit supply, thus testing was compromised in those weeks. The overall testing rate for ILI and sARI cases are 94.8% and 100%, respectively, for pregnant women and 77.6% and 30.6%, respectively, for infants under 6 months.

Fig 2 shows the weekly number of virus positives detected in the two populations. In general, positive cases were detected mainly from January to February, coinciding with the high activities of both ILI and sARI. All three targeted viruses were detected during the 2013/14 season, while influenza B virus was not detected during the 2014/15 season. This was quite consistent with data from the national ILI surveillance, except that RSV was not detected during the 2013/14 season (S1 Fig). There were periods of limited point-of-care test kit availability at the beginning of both seasons and early December in the 2014/15 season. Hence, virus testing was compromised during those periods.

### Incidence rates of ILI, sARI and virus positives for pregnant women

We individually enrolled 1,260 pregnant women and they were followed for a total of 120,887 person-days. The population characteristics were quite different for the two seasons (S1 Table). Briefly, the 2014/15 season cohort tend to be more educated, employed, overweight, enrolled at an earlier gestational age, and did not have co-morbidity; though these differences may possibly be due to chance. The median age at enrolment was 27 years (range: 16–44) and 43.0% were enrolled during the first trimester. Seven pregnant women refused to join the study. None had received the influenza vaccine.

A total of 174 ILI episodes (13.8%) were detected from 160 pregnant women. There were 12 and 2 women with 2 and 3 ILI episodes respectively, during the same influenza season. Among the ILI cases with recorded symptom resolution dates (85.6%), the mean interval to resolution was 8.1 days (range: 3–20). The overall IR for ILI was 11.8 per 1,000 person-days (95% C.I: 11.2–12.4) (Table 1).

**Table 1:** Incidence rates (IRs) of ILI and sARI cases detected for pregnant women, stratified by trimester stage and for the overall cohort.

Trimester stage	ILI		sARI	
	No. of cases	IR per 1,000 person-days (95% CI)	No. of cases	IR per 1,000 person-days (95% CI)
1st	35	29.0 (25.9–32.5)	1	0.9 (0.2–5.2)
2nd	83	11.7 (10.9–12.6)	1	0.1 (0.03–0.8)
3rd	56	8.70 (7.96–9.51)	0	0 (0.0–0.4)
Overall	174	11.8 (11.2–12.4)	2	0.1 (0.04–0.4)

Among all tested ILI cases (n = 165), 26 (15.8%) tested positive for influenza A, 2 (1.2%) for influenza B, and 4 (2.4%) for RSV. This gives an overall IR of 1.7 per 1,000 person-days (95% C.I: 1.5–1.9) for influenza A, 0.1 per 1,000 person-days (95% C.I: 0.1–0.2) for influenza B, and 0.3 per 1,000 person-days (95% C.I: 0.2–0.4) for RSV (Table 2). The majority of samples (96.0%) were tested within 5 days after symptom onset. During the 2014/15 season, 2 women tested positive for both influenza A and RSV from separate ILI episodes.

**Table 2:** Incidence rates (IRs) of influenza A, influenza B and RSV detected for pregnant women, stratified by trimester stage and for the overall cohort.

Trimester stage	Influenza A		Influenza B		RSV	
	No. of cases	IR per 1,000 person-days (95% CI)	No. of cases	IR per 1,000 person-days (95% CI)	No. of cases	IR per 1,000 person-days (95% CI)
1st	6	4.4 (3.3–5.9)	1	0.9 (0.5–1.6)	1	0.8 (0.4–1.5)
2nd	15	2.1 (1.7–2.5)	0	0	1	0.1 (0.1–0.3)
3rd	5	0.7 (0.5–1.0)	1	0.1 (0.1–0.3)	2	0.3 (0.2–0.5)
Overall	26	1.7 (1.5–1.9)	2	0.1 (0.1–0.2)	4	0.3 (0.2–0.4)

Two sARI cases were also detected, giving an overall sARI incidence rate of 0.1 per 1,000 person-days (95% C.I: 0.04–0.4). One case tested negative in the 2014/15 season during her second trimester, and another was positive for influenza A during the 2013/14 season during her first trimester. No virus co-detection and deaths were observed during the study period.

IRs for ILI and influenza A were the lowest in the third trimester (Tables 1 and 2). Sensitivity analysis using total person-days only in January and February also revealed the same trend as the main result. This analysis was done because all influenza A positive cases were detected only in the latter months. Similar trend was also observed in the risk factor analysis for both ILI (Table 3) and influenza A (Table 4). When compared to the first trimester, pregnant women in the third trimester were 78% less likely to have influenza A detected, after adjusting for age and presence of prior pregnancy [Adjusted hazard ratio (Adj. HR): 0.22 (95% C.I: 0.07–0.73)]. Pregnant women were also 50% [Adj. HR: 0.50 (95% C.I: 0.34–0.75)] and 63% [Adj. HR: 0.37 (95% C.I: 0.25–0.55)] less likely to develop an ILI episode in the second and third trimesters, respectively, when compared to the first trimester. In addition, pregnant women who have any co-morbidity were 43% more likely to develop an ILI episode [Adj. HR: 1.43 (95% C.I: 1.06–1.94)].

**Table 3:** Risk factor analysis results when comparing ILI cases and non-ILI among pregnant women. Co-variates were chosen based on initial univariate analyses (S3 Table). Estimates in bold indicate statistically significant co-variates.

		Hazard ratio (95% C.I)	
Pregnant women and ILI episodes		Un-adjusted	Adjusted
Trimester of symptom onset	1st	1 (ref)	1 (ref)
	2nd	**0.52 (0.35–0.78)**	**0.50 (0.34–0.75)**
	3rd	**0.38 (0.25–0.56)**	**0.37 (0.25–0.55)**
Highest education attained	College or higher	1 (ref)	1 (ref)
	High school or lower	0.69 (0.45–1.04)	0.92 (0.54–1.56)
	Employed ^#^	1 (ref)	1 (ref)
Employment status	Unemployed	0.86 (0.59–1.26)	1.00 (0.67–1.50)
	Student	0.54 (0.27–1.08)	0.75 (0.31–1.81)
	A	1 (ref)	1 (ref)
FGP	B	1.46 (0.94–2.26)	1.44 (0.91–2.28)
	C	1.34 (0.87–2.06)	1.30 (0.85–1.99)
	D	1.06 (0.65–1.74)	1.13 (0.69–1.83)
Age at enrolment		1.02 (1.00–1.05)	1.00 (0.97–1.03)
Has any co-morbidity ^^		**1.41 (1.04–1.91)**	**1.43 (1.06–1.94)**
Classified as high risk pregnancy		1.28 (0.94–1.73)	1.15 (0.76–1.80)
Has prior pregnancy		1.32 (0.92–1.89)	1.17 (0.76–1.80)
Has kindergarten-age child (2 – 5yrs) in household		1.19 (0.88–1.60)	1.11 (0.81–1.51)

^#^ Includes employment in power stations, coal mining, agriculture, offices, schools and healthcare

^^^^ Missing value for two participants

**Table 4:** Risk factor analysis results when comparing influenza A positive cases and non-ILI among pregnant women. Co-variates were chosen based on initial univariate analyses. Estimates in bold indicate statistically significant co-variates.

		Hazard ratio (95% C.I)	
Pregnant women and influenza A cases		Un-adjusted	Adjusted
Trimester of symptom onset	1st	1 (ref)	1 (ref)
	2nd	0.62 (0.24–1.59)	0.63 (0.24–1.62)
	3rd	**0.22 (0.07–0.72)**	**0.22 (0.07–0.73)**
Age at enrolment		1.02 (0.96–1.09)	0.98 (0.90–1.07)
Has prior pregnancy		3.09 (0.93–10.3)	3.45 (0.94–12.70)

### ILI and sARI incidence, and virus positivity rates for infants under 6 months

We individually enrolled 1,304 infants under 6 months and they were followed for a total of 122,344 person-days. They were mainly healthy, whereby 88.4% were born full term, 2.9% had low birthweight, and 3.5% were born with a certain birth defect. Their baseline characteristics were similar for both seasons, except that there were significantly more full term infants in the 2014/15 season (S2 Table). The median age at enrolment was 12 days (range: 0–167). During the 2013/14 season, 99.9% were breastfed for 6 months.

A total of 246 ILI episodes (18.9%) were detected from 201 infants under 6 months. There were 37, 7 and 1 infants with 2, 3, and 4 ILI episodes, respectively, during the same influenza season. Among the ILI cases with recorded symptom resolution dates (82.2%), the mean interval to resolution was 7.5 days (range: 1–14). The overall ILI IR was 15.2 per 1,000 person-days (95% C.I: 14.5–15.8) (Table 5). From all tested ILI cases (n = 191), we detected 15 (7.9%) influenza A, 3 (1.6%) influenza B and 8 (4.2%) RSV cases. Most samples were tested within 5 days after symptom onset (98.8%). During the 2013/14 season, we detected RSV twice (on separate occasions) from one female infant of low birthweight and did not result in sARI hospitalization.

**Table 5:** Incidence rates (IRs) of ILI and sARI cases for infant under 6 months, stratified by age groups and for the overall cohort.

Age group (weeks)	ILI		sARI	
	No. of cases	IR per 1,000 person-days (95% CI)	No. of cases	IR per 1,000 person-days (95% CI)
0–7.9	46	9.10 (8.21–10.1)	80	21.3 (20.0–22.8)
8–15.9	69	10.9 (10.0–11.9)	72	15.7 (14.7–16.9)
16–24	131	26.8 (25.2–28.5)	103	25.8 (24.2–27.4)
Overall	246	15.2 (14.5–15.8)	255	20.5 (19.7–21.3)

A total of 255 sARI hospitalized cases (19.6%) were detected from 218 infants under 6 months, giving an overall sARI IR of 20.5 per 1,000 person-days (95% C.I: 19.7–21.3) (Table 5). There were 30 and 8 infants who were hospitalized for sARI twice and thrice, respectively, during the same influenza season. Also, 36 (14.1%) of them had prior visit to the FGP for the same illness episode. The mean length of hospital stay was 8.9 days (range: 0–33). For all sARI cases, 74.3% were admitted within 5 days after symptom onset. A total of 23 (18.3%) and 55 (42.6%) cases were tested during the 2013/14 and 2014/15 season, respectively. This gives an overall sARI testing rate of 30.6%. During the 2013/14 season, 4 (17.4%) tested positive for RSV and 2 (8.7%) for influenza A while 13 (23.6%) RSV and no influenza A cases were detected during the 2014/15 season. No influenza B positive sARI cases were detected from either season. No virus co-detection and deaths were also observed during the study period.

Overall, we detected a total of 17 (6.3%) influenza A, 3 (1.1%) influenza B, and 25 (9.3%) RSV cases among the tested ILI and sARI samples (n = 269) (Table 6). Among the total positive cases, 2 (11.8%) influenza A and 17 (68.0%) RSV cases were detected among hospitalized cases with sARI. After stratification by age group, 16–24 weeks old infants had the highest IR for ILI [26.8 per 1,000 person-days (95% C.I: 25.2–28.5)] and sARI [25.8 per 1,000 person-days (95% C.I: 24.2–27.4)] (Table 5). The same trend was also observed in the positivity rates of influenza A and RSV (Table 6). The increase in the RSV positivity rate started from the 8–15.9 weeks old group. Also, sARI cases were 1.4 times more likely to be male, when compared to non-sARI [Adj. HR: 1.40 (95% C.I: 1.07–1.83)] (Table 7).

**Table 6:** Total positive case numbers of influenza A, influenza B and RSV and also those hospitalized for sARI, stratified by age groups and for the overall cohort.

Age group (weeks)	Total samples tested	Influenza A		Influenza B		RSV	
		Total cases (%)	sARI cases only (%)	Total cases (%)	sARI cases only (%)	Total cases (%)	sARI cases only (%)
0–7.9	54	2 (3.7)	0 (0.0)	1 (1.9)	0	3 (5.6)	3 (5.6)
8–15.9	79	2 (2.5)	1 (1.3)	0 (0.0)	0	8 (10.1)	7 (8.9)
16–24	136	13 (9.6)	1 (0.7)	2 (1.5)	0	14 (10.3)	7 (5.1)
Overall	269	17 (6.3)	2 (0.7)	3 (1.1)	0	25 (9.3)	17 (6.3)

**Table 7:** Risk factor analysis results when comparing sARI cases and non-sARI among infants under 6 months. Co-variates were chosen based on initial univariate analyses (S4 Table). Estimates in bold indicate statistically significant co-variates.

	Hazard ratio (95% C.I)	
Infant < 6 months and sARI cases	Un-adjusted	Adjusted
Age group (weeks)		
0–7.9	1.05 (0.78–1.42)	1.03 (0.76–1.39)
8–15.9	0.76 (0.55–1.04)	0.75 (0.55–1.04)
16–24	1 (ref)	1 (ref)
Gender		
Female	1 (ref)	1 (ref)
Male	**1.42 (1.09–1.85)**	**1.40 (1.07–1.83)**
Has birth defect^		
No	1 (ref)	1 (ref)
Yes	1.40 (0.86–2.28)	1.09 (0.57–2.11)
Gestational age at delivery		
Full term	1 (ref)	1 (ref)
Preterm	2.64 (1.28–5.44)	2.26 (0.83–6.16)
Early term	1.10 (0.73–1.66)	1.10 (0.71–1.70)

^^^ Missing value for one participant

## Discussion

We prospectively followed a cohort of unvaccinated pregnant women and infants under 6 months to actively detect acute respiratory illnesses for two consecutive influenza seasons. Among pregnant women, we observed low overall IR for influenza A. Both ILI and influenza A IR were the lowest in the third trimester. There were also few sARI and influenza A positive hospitalized cases. Also, pregnant women with co-morbidity have about 1.4 times higher risk of developing an ILI episode. Among infants under 6 months, we observed moderately high ILI and sARI IRs. Older infants (16–24 weeks old) had higher ILI IR and positivity rates (of influenza A and RSV), while younger male infants (0–7.9 weeks old) had higher sARI IR. Among all influenza A and RSV positive infant cases, 11.8% and 68.0% were respectively identified among hospitalized cases with sARI.

One study in the United States [35] reported both ILI and sARI IR for pregnant women in all three trimesters and they observed high IRs, especially for sARI. Both ILI and sARI IRs in the study are also higher than that previously reported for the reproductive-age population (15–49 years) in Baganuur [36] and Mongolia [25], though it could possibly be due to active case search in the study. Expanding this study to include non-pregnant women of reproductive-age would allow us to see any impact of pregnancy in acquiring ILI and sARI. These Mongolian studies also reported incidence rates for young children, though aggregates of 0–11 months was used. Here, ILI IR in this study is higher than that reported in Baganuur [36]. Relatively high infant ILI IRs were also previously reported from Bangladesh [13] and the United States [14]. Nevertheless, caution should be taken for interpreting these direct comparisons due to differences in case detection methods, study season, and health-seeking tendencies, that is between women with and without pregnancy, as well as between younger and older infants.

The same study from Mongolia [25] also reported influenza positive rates by age group. The overall influenza positive rates for both ILI and sARI cases were lowest in the 0–11 month age group and highest in the reproductive age groups (16–44 years). Our study findings for infants under 6 months were consistent with the former, as more RSV positives than influenza were detected. The latter was also consistent with this study as pregnant women were mainly positive for influenza A. RSV infection was also documented for young working adults [37], though the attack rate was lower and symptom severity was milder than those of infants. Despite the fact that influenza B can affect people of all ages, only few influenza B cases were detected in both pregnant women and infants, presumably reflecting the circulation of influenza virus in the community.

Both IR and adjusted HR of influenza A were lowest for pregnant women in the third trimester, which could be due to differences in virus exposure at each trimester stage. Studies in the United States have shown that women in the third trimester tend to engage in lesser leisure activities [38] and spend more time at home [39]. Although it is uncertain whether both tendencies are also observed among Mongolians, it can lead to less interaction with others in the community, thus decreasing the probability of acquiring respiratory infections. Notably, we also observed the same finding in the risk factor analysis for ILI, after accounting for the presence of co-morbidity as well as possible transmission in the household (presence of kindergarten-age child) and workplace (employment status). This suggests that settings other than household and workplace may contribute to a certain risk of acquiring ILI and possibly influenza A infections. However, our finding contradicts with that in a previous study which showed high IR of influenza A in the third trimester [40]. Experimental studies have reported that the immune system switch to defensive mode during pregnancy [41]. Defensive immune responses (via phagocytic cells and α-defensins 1–3) are elevated throughout the pregnancy period [41]. Estrogen and progesterone levels, which surge from the first trimester [42], further strengthens this by stimulating anti-inflammatory responses [43]. This means that the immune system is capable of preventing the establishment of viral infections as pregnancy progresses, and that there is little immunological basis for increasing susceptibility to influenza infection [44]. Hence, our study finding is in fact more consistent to what is known with the immunological changes during pregnancy.

We also observed low sARI IR among pregnant women and only one was positive for influenza A. This observation is in line with some studies [45, 46], but there are also many reports of increased influenza-associated hospitalization rates during the inter-pandemic periods [5, 6, 35, 47–49]. Our study findings could firstly be due to the relatively mild influenza seasons. Antigenic changes to the circulating strains seemed limited during both seasons under study, as the WHO-recommended vaccine strains were the same [50]. Thus, the enrolled pregnant women may have already had the infection to a similar strain prior to current pregnancy. Secondly, it could also be the low sample size of this study, which makes it difficult to detect severe illnesses that occur at a low rate.

We also found that pregnant women with co-morbidity had 1.4 times higher risk of developing an ILI episode, a finding which is consistent with previous studies [35]. Although this HR estimate was for both seasons, additional analysis for each season revealed significance only for the 2014/15 season. When compared to the 2013/14 season, the 2014/15 season had a lower proportion of pregnant women with co-morbidity enrolled and influenza A activity was also higher. This thus suggests that having co-morbidity is in fact a risk factor of developing ILI symptoms associated with influenza among pregnant women.

The ILI and sARI IR for infants under 6 months were the highest among the 16–24 weeks old group and similar trend was also observed in the positivity rates of both influenza A and RSV. In fact, 100% and 82.4% of the influenza A and RSV positive sARI cases, respectively, were found in those aged 8 weeks or older. Notably, this finding for RSV is consistent with that in Kenya [51]. This could be explained by the waning of passively-acquired maternal antibodies. Infants under 6 months who had acquired maternal antibodies against influenza [52, 53] and RSV [54] were reported to be protected from severe illness and had delayed symptom onset. Also, protection from maternal antibodies tends to wane after the first 2–3 months of life. Previous studies reported the half-life of passively-acquired maternal antibodies to be 21–53 days against influenza [52, 53, 55, 56] and 26–78 days against RSV [57–59].

sARI IR was also high among the 0–7.9 weeks old infants, giving the impression of low IR among the 8–15.9 weeks old. However, this could be falsely elevated due to two reasons. First, young infants tend to exhibit signs of breathing difficulties, regardless of infection severity. Thus, parents/guardians tend to bring them directly to the hospital for immediate care. In fact, only 38 sARI cases (14.8%) were referred to the hospital from the FGP; hence suggesting a small number of ‘true’ sARI cases. Second, Mongolia follows the integrated management of childhood illness (IMCI) guidelines, whereby infants < 2 months old with fever are to be directly admitted to the hospital as a sARI case [60]. Hence, the high tendency to seek healthcare at the hospital and the implementation of the IMCI guidelines could explain the high sARI IR among younger infants. This could also be one reason why the overall IR for sARI was higher than that of ILI.

We also observed that 68.0% of RSV-positives detected were hospitalized with sARI, which is highly disproportionate when compared to that of influenza A (11.8%). This tendency is consistent with previous studies on hospitalized infants in the United States [9, 10, 61] and Thailand [62]. This data indicates that RSV caused more severe illness that required hospitalization among infants under 6 months in Baganuur. As the testing rate was low, particularly for sARI cases, it means that the actual proportion of hospitalized cases for both viruses could be much higher.

Infant sARI cases were 1.4 times more likely to be males, a trend that is consistent with previous reports [63] and also seen with RSV-associated hospitalizations [64]. When compared to females, young male infants tend to have reduced airway function [65, 66] and also tend to be more susceptible to infection [67]. In addition, all the RSV positive sARI cases were born full term. This means that RSV disease can also affect healthy infants, a finding in line with previous studies [10, 68].

Some study limitations need to be addressed. First, point-of-care test was the main diagnostic method used, which could lower the sensitivity of virus detection. A meta-analysis on influenza test kit evaluation studies reported a pooled sensitivity and specificity of 62.3% and 98.2%, respectively [69]. Thus, the incidence and positivity rates reported in this study are an underestimate, possibly by about 40%. For RSV, antigen detection methods are known to be considerably less sensitive particularly for adults [70]. We used point-of-care test kits mainly due to the limited laboratory capacity to test samples with real-time PCR. It also allowed us to simultaneously test for RSV, as the local laboratory only tests routinely for influenza. In order to ensure higher viral loads in the samples taken, efforts were made to identify cases within 5 days of symptom onset, and to test them as soon as possible upon identification. Second, there was a limited supply of point-of-care test kits in some periods of the study, resulting in a low overall testing rate for the sARI cases among infants (30.6%). This remains to be a major limitation even though we have improved the sARI testing rate from 18.1% in the 2013/14 season to 42.6% in the 2014/15 season. Hence, the positive rates reported for the infants under 6 months are an underestimate. Third, as a consequence of using point-of-care test kits, we also could not provide the IR by influenza subtype and RSV genotype. Fourth, we did not assess the use of non-pharmaceutical interventions, such as hand washing and facemask usage, in our study population. This could possibly affect the risk factor analysis results. Fifth, we did not detect enough virus-positive cases for both seasons to assess their risk factors. This could merely reflect the low influenza and RSV activities in this community and thus caution must be taken to extrapolate our findings to other areas in Mongolia. Sixth, we used the dates of follow-up and hospitalization to determine the symptomatic period for ILI and sARI IR calculations, respectively. These dates do not strictly reflect on each clinical course and may affect the calculated IRs. Lastly, we collected household demographic information among infants only for the 2014/15 season. As a result, these data were excluded from the risk factor analyses for both seasons.

In conclusion, we reported the results of a prospective, observational open cohort study conducted in a community setting for two targeted high-risk groups, during two consecutive influenza seasons in Mongolia. We observed a low overall influenza A burden for pregnant women in terms of incidence and hospitalization rates, though underestimation was likely due to point-of-care tests used. One surprising finding is the low influenza A incidence rate among the third trimester women, despite their particularly high risk of developing influenza-related severe illness. While for infants under 6 months, the incidence of ILI and sARI was moderately high. An important note here is the high number of RSV positives who got hospitalized when compared to that of influenza A, despite the low testing rate. Including additional data from subsequent influenza seasons would help to ascertain these findings. Despite its limitations, our study findings add into the currently limited knowledge on the burden of seasonal influenza for both groups. This is also the first study of its kind in Mongolia, a developing country where maternal and child health is an important health topic.

## Supporting Information

S1 Dataset Dataset for pregnant women, for incidence rate calculation and risk factor analysis. (XLSX)

S2 Dataset Dataset for infants under 6 months, for incidence rate calculation and risk factor analysis. (XLSX)

S1 Fig The weekly number of positive cases detected in Baganuur district for 2013/14 (left) and 2014/15 (right) seasons, based on the national ILI surveillance [31]. The bars show the number of positive cases for influenza A(H3N2) [brown], influenza A(H1N1)pdm09 [pink], influenza B [blue] and RSV [green].
(TIF)

S1 Table Baseline characteristics of the pregnant women cohort and its comparison between two seasons. (DOCX)

S2 Table Baseline characteristics of the infants under 6 months cohort and its comparison for the each season. (DOCX)

S3 Table Characteristics of the ILI cases and non-ILI for the pregnant women cohort. (DOCX)

S4 Table Characteristics of the (a) ILI cases and non-ILI, and (b) sARI cases and non-sARI for the infants under 6 months cohort. (DOCX)
