# Supplementary figures and images for: Burden of Influenza and Respiratory Syncytial Virus Infection in Pregnant Women and Infants Under 6 Months in Mongolia: A Prospective Cohort Study
Source: PLoS One. 2016 Feb 5;11(2):e0148421. doi: 10.1371/journal.pone.0148421 (PMC4746066; doi:10.1371/journal.pone.0148421)

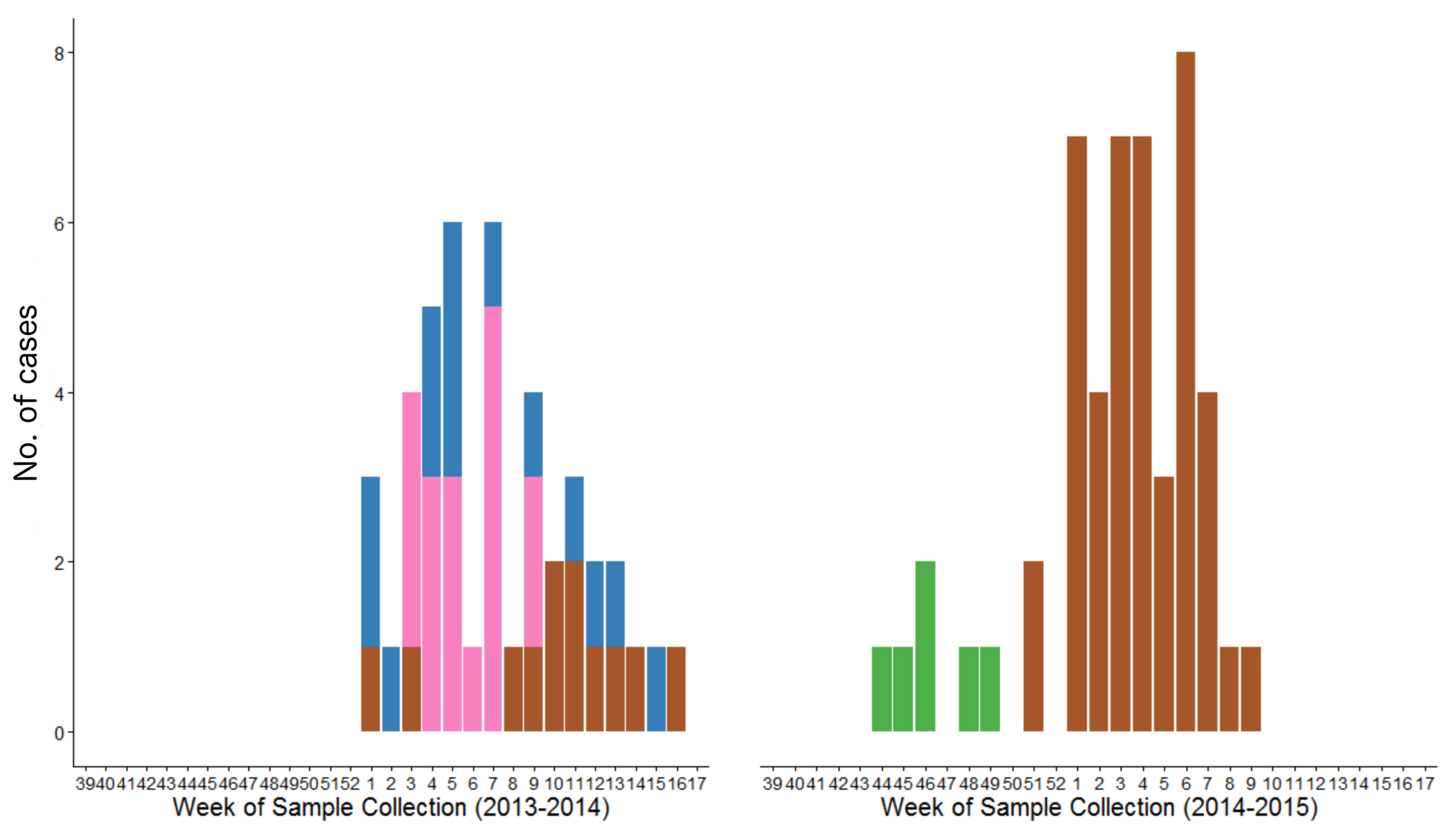

Supplement: S1 Fig — The bars show the number of positive cases for influenza A(H3N2) [brown], influenza A(H1N1)pdm09 [pink], influenza B [blue] and RSV [green]. (TIF) [file pone.0148421.s003.tif]
