# Supplementary material for: Burden of Influenza and Respiratory Syncytial Virus Infection in Pregnant Women and Infants Under 6 Months in Mongolia: A Prospective Cohort Study
Source: PLoS One. 2016 Feb 5;11(2):e0148421. doi: 10.1371/journal.pone.0148421 (PMC4746066; doi:10.1371/journal.pone.0148421)
Supplement: S1 Table — (DOCX) [file pone.0148421.s004.docx]

**S1 Table.** Baseline characteristics of the pregnant women cohort and its comparison between two seasons.

| **Population characteristic** | | **Total cohort (%)** | **2013/14 season (%)** | **2014/15 season (%)** | ***p*-value** |
| --- | --- | --- | --- | --- | --- |
| No. of pregnant women enrolled | | 1260 | 643 (51.0) | 617 (49.0) |  |
| Age at enrolment (years) | Median ± sd | 27.4 ± 6.1 | 27.0 ± 6.1 | 28.0 ± 6.0 | 0.07 |
|  | Range | 16 - 44 | 16 - 43 | 16 - 44 |  |
| Educated at high-school level or lower | | 278 (22.1) | 212 (33.0) | 66 (10.7) | <0.001* |
| Employment status | Employed ^#^ | 825 (65.5) | 381 (59.3) | 444 (72.0) |  |
|  | Unemployed | 326 (25.9) | 193 (30.0) | 133 (21.5) | <0.001* |
|  | Student | 109 (8.6) | 69 (10.7) | 40 (6.5) |  |
| BMI category ^ | Underweight (< 18.5) | 51 (4.1) | 22 (3.4) | 29 (4.7) |  |
|  | Normal (18.5-24.9) | 845 (67.1) | 471 (73.4) | 374 (60.6) |  |
|  | Overweight (25-29.9) | 296 (23.5) | 126 (19.6) | 170 (27.6) | <0.001* |
|  | Obese (≥ 30) | 67 (5.3) | 23 (3.6) | 44 (7.1) |  |
| ***Household characteristics*** | |  |  |  |  |
| Type of household structure ^ | Apartment | 544 (43.2) | 297 (46.3) | 247 (40.1) |  |
|  | Ger | 549 (43.6) | 236 (36.7) | 313 (50.7) | <0.001* |
|  | Private house | 166 (13.2) | 109 (17.0) | 57 (9.2) |  |
| Household size | 1 - 2 | 173 (13.7) | 82 (12.8) | 91 (14.7) |  |
|  | 3 - 4 | 693 (55.0) | 353 (54.9) | 340 (55.1) | 0.5 |
|  | 5 and above | 394 (31.3) | 208 (32.3) | 186 (30.2) |  |
| Young child present (< 2yrs) | | 135 (10.7) | 61 (9.5) | 74 (12.0) | 0.18 |
| Kindergarten-age child present (2 - 5yrs) | | 491 (39.0) | 244 (37.9) | 247 (40.0) | 0.48 |
| School-age child present (6 - 17yrs) | | 638 (50.6) | 325 (50.5) | 313 (50.7) | 0.99 |
| ***Obstetrics characteristics*** | |  |  |  |  |
| FGP consulted | A | 296 (23.5) | 159 (24.7) | 137 (22.2) |  |
|  | B | 309 (24.5) | 157 (24.4) | 152 (24.6) |  |
|  | C | 404 (32.1) | 195 (30.3) | 209 (33.9) | 0.51 |
|  | D | 251 (19.9) | 132 (20.6) | 119 (19.3) |  |
| Gestational age at enrolment | Median ± sd | 16.1 ± 9.6 | 19.4 ± 10.0 | 13.0 ± 8.4 | <0.001* |
|  | Range | 1.7 - 41.0 | 1.7 - 41 | 2.6 - 38.7 |  |
| Has prior pregnancy | | 879 (69.8) | 435 (67.7) | 444 (72.0) | 0.11 |
| Has any co-morbidity ^^ | | 440 (34.9) | 323 (50.2) | 117 (19.0) | <0.001* |
| Classified as high risk pregnancy | | 601 (47.7) | 264 (41.1) | 337 (54.6) | <0.001* |
| ^#^ Includes employment in power stations, coal mining, agriculture, offices, schools and healthcare | | | | |  |
| ^ Missing value for one participant  ^^ Missing value for two participants  * *p*-value of < 0.05 is considered to be significant | | | | | |
