# Supplementary material for: Burden of Influenza and Respiratory Syncytial Virus Infection in Pregnant Women and Infants Under 6 Months in Mongolia: A Prospective Cohort Study
Source: PLoS One. 2016 Feb 5;11(2):e0148421. doi: 10.1371/journal.pone.0148421 (PMC4746066; doi:10.1371/journal.pone.0148421)
Supplement: S2 Table — (DOCX) [file pone.0148421.s005.docx]

**S2 Table.** Baseline characteristics of the infants under 6 months cohort and its comparison between two seasons.

| **Population characteristics** | | **Total cohort (%)** | **2013/14 season (%)** | **2014/15 season (%)** | ***p*-value** |
| --- | --- | --- | --- | --- | --- |
| No. of infants < 6 months enrolled | | 1304 | 692 (53.0) | 612 (47.0) |  |
| Male gender | | 673 (51.6) | 364 (52.6) | 309 (50.5) | 0.48 |
| Age at enrolment (days) | Median ± sd | 12.0 ± 50.9 | 13.0 ± 51.7 | 9.0 ± 49.9 | 0.06 |
|  | Range | 0 - 167 | 0 - 167 | 0 - 167 |  |
| ***Newborn characteristics*** | |  |  |  |  |
| Birth defect present ^ | | 46 (3.5) | 31 (4.5) | 15 (2.5) | 0.051 |
| Had low birthweight | | 38 (2.9) | 25 (3.6) | 13 (2.1) | 0.14 |
| Term of pregnancy | Preterm (< 37 wks) | 9 (0.7) | 6 (0.9) | 3 (0.5) |  |
|  | Early term (37-38 wks) | 142 (10.9) | 111 (16.0) | 31 (5.1) | <0.001* |
|  | Full term (≥ 39 wks) | 1153 (88.4) | 575 (83.1) | 578 (94.4) |  |
| FGP consulted | A | 318 (24.4) | 177(25.6) | 141 (23.1) |  |
|  | B | 360 (27.6) | 199 (28.8) | 161 (26.3) | 0.28 |
|  | C | 371 (28.4) | 192 (27.7) | 179 (29.2) |  |
|  | D | 255 (19.6) | 124 (17.9) | 131 (21.4) |  |

^^ Missing value for one participant^

^*^ *^p^*^-value of < 0.05 is considered to be significant^
