# Supplementary material for: Burden of Influenza and Respiratory Syncytial Virus Infection in Pregnant Women and Infants Under 6 Months in Mongolia: A Prospective Cohort Study
Source: PLoS One. 2016 Feb 5;11(2):e0148421. doi: 10.1371/journal.pone.0148421 (PMC4746066; doi:10.1371/journal.pone.0148421)
Supplement: S3 Table — (DOCX) [file pone.0148421.s006.docx]

**S3 Table.** Characteristics of the ILI cases and non-ILI for the pregnant women cohort.

| **Population characteristics** | | **ILI (%)** | **non-ILI (%)** | ***p*-value** |
| --- | --- | --- | --- | --- |
| No. of pregnant women enrolled | Total | 174 (13.8) | 1100 (87.3) |  |
|  | 2013/14 season | 110 (63.2) | 542 (49.3) | <0.001* |
|  | 2014/15 season | 64 (36.8) | 558 (50.7) |  |
| Age at enrolment (years) | Median ± sd | 29 ± 6.0 | 27 ± 6.0 | 0.01** |
|  | Range | 17 - 44 | 16 - 44 |  |
| Educated at high-school level or lower | | 26 (14.9) | 253 (23.0) | 0.02** |
| Employment status | Employed ^#^ | 127 (73.0) | 707 (64.3) | 0.04** |
|  | Unemployed | 39 (22.4) | 292 (26.5) |  |
|  | Student | 8 (4.6) | 101 (9.2) |  |
| BMI category ^ | Underweight (< 18.5) | 8 (4.6) | 44 (4.0) | 0.54 |
|  | Normal (18.5-24.9) | 122 (70.5) | 731 (66.5) |  |
|  | Overweight (25-29.9) | 37 (21.4) | 263 (23.9) |  |
|  | Obese (≥ 30) | 6 (3.5) | 62 (5.6) |  |
| ***Household characteristics*** | |  |  |  |
| Type of household structure ^ | Apartment | 83 (47.7) | 468 (42.6) | 0.2 |
|  | Ger | 75 (43.1) | 481 (43.8) |  |
|  | Private house | 16 (9.2) | 150 (13.6) |  |
| Household size | 1 - 2 | 21 (12.1) | 153 (13.9) | 0.78 |
|  | 3 - 4 | 99 (56.9) | 603 (54.8) |  |
|  | 5 and above | 54 (31.0) | 344 (31.3) |  |
| Young child present (< 2yrs) | | 14 (8.0) | 122 (11.1) | 0.29 |
| Kindergarten-age child present (2 - 5yrs) | | 78 (44.8) | 417 (37.9) | 0.09** |
| School-age child present (6 - 17yrs) | | 85 (48.9) | 560 (50.9) | 0.67 |
| ***Obstetrics characteristics*** | |  |  |  |
| Trimester at enrolment | 1st (0 - 13 weeks) | 101 (58.0) | 452 (41.1) | <0.001** |
|  | 2nd (14 - 26 weeks) | 59 (33.9) | 374 (34.0) |  |
|  | 3rd (≥ 27 weeks) | 14 (8.1) | 274 (24.9) |  |
| Gestational age at enrolment | Median ± sd | 12.4 ± 7.7 | 16.6 ± 9.7 | <0.001** |
|  | Range | 2.0 - 34.0 | 1.7 - 41.0 |  |
| FGP consulted | A | 31 (17.8) | 266 (24.2) | 0.07** |
|  | B | 52 (29.9) | 263 (23.9) |  |
|  | C | 63 (36.2) | 348 (31.6) |  |
|  | D | 28 (16.1) | 223 (20.3) |  |
| Has prior pregnancy | | 134 (77.0) | 757 (68.8) | 0.04** |
| Has any co-morbidity ^^ | | 77 (44.3) | 371 (33.7) | 0.006** |
| Classified as high risk pregnancy | | 98 (56.3) | 511 (46.5) | 0.02** |
| ^#^ Includes employment in power stations, coal mining, agriculture, offices, schools and healthcare | | | |  |
| ^ Missing value for one participant | | | | |
| ^^ Missing value for two participants  * *p*-value of < 0.05 is considered to be significant | | | |  |
| ** Variable was included in Cox PH model | | | |  |
