# Supplementary material for: Burden of Influenza and Respiratory Syncytial Virus Infection in Pregnant Women and Infants Under 6 Months in Mongolia: A Prospective Cohort Study
Source: PLoS One. 2016 Feb 5;11(2):e0148421. doi: 10.1371/journal.pone.0148421 (PMC4746066; doi:10.1371/journal.pone.0148421)
Supplement: S4 Table — (DOCX) [file pone.0148421.s007.docx]

**S4 Table.** Characteristics of the (a) ILI cases and non-ILI, and (b) sARI cases and non-sARI for the infants under 6 months cohort.

| **Population characteristics** | | **ILI cases (%)** | **Non-ILI (%)** | ***p*-value** | **sARI cases (%)** | **Non-sARI (%)** | ***p*-value** |
| --- | --- | --- | --- | --- | --- | --- | --- |
| No. of infants < 6 months enrolled | Total | 246 (18.9) | 1103 (84.6) |  | 255 (19.6) | 1088 (83.4) |  |
|  | 2013/14 season | 129 (52.4) | 583 (52.9) | 0.96 | 126 (49.4) | 580 (53.3) | 0.35 |
|  | 2014/15 season | 117 (47.6) | 520 (47.1) |  | 129 (50.6) | 508 (46.7) |  |
| Male gender | | 125 (50.8) | 571 (51.8) | 0.84 | 149 (58.4) | 551 (50.6) | 0.03** |
| Age at enrolment (days) | Median ± sd | 21 ± 34.3 | 11 ± 53.1 | <0.001** | 8 ± 39.5 | 12 ± 52.6 | 0.01** |
|  | Range | 1 - 144 | 0 - 167 |  | 0 - 155 | 0 - 167 |  |
| ***Newborn characteristics*** | |  |  |  |  |  |  |
| Birth defect present ^ | | 10 (4.0) | 39 (3.5) | 0.83 | 14 (5.5) | 32 (2.9) | 0.06** |
| Had low birthweight | | 6 (2.4) | 33 (3.0) | 0.83 | 11 (4.3) | 27 (2.5) | 0.17 |
| Term of pregnancy | Preterm (< 37 weeks) | 0 (0.0) | 9 (0.8) |  | 5 (2.0) | 4 (0.4) | 0.03** |
|  | Early term (37-38 weeks) | 25 (10.2) | 120 (10.9) | 0.49 | 10 (3.9) | 41 (3.8) |  |
|  | Full term (≥ 39 weeks) | 221 (89.8) | 974 (88.3) |  | 240 (94.1) | 1043 (95.8) |  |
| FGP consulted | A | 50 (20.3) | 278 (25.2) | 0.002** | 63 (24.7) | 268 (24.6) | 0.77 |
|  | B | 88 (35.8) | 291 (26.4) |  | 64 (25.1) | 302 (27.8) |  |
|  | C | 76 (30.9) | 308 (27.9) |  | 73 (28.6) | 309 (28.4) |  |
|  | D | 32 (13.0) | 226 (20.5) |  | 55 (21.6) | 209 (19.2) |  |
| ***Household characteristics (For 2014/15 season only)*** | | |  |  |  |  |  |
| Type of household structure | Apartment | 30 (25.6) | 240 (46.2) | <0.001** | 77 (59.7) | 206 (40.6) | <0.001** |
|  | Ger | 71 (60.7) | 178 (34.2) |  | 38 (29.4) | 198 (39.0) |  |
|  | Private house | 16 (13.7) | 102 (19.6) |  | 14 (10.9) | 104 (20.5) |  |
| Household size | 1 - 2 | 3 (2.6) | 18 (3.4) | 0.78 | 7 (5.4) | 15 (3.0) | 0.20 |
|  | 3 - 4 | 55 (47.0) | 225 (43.3) |  | 62 (48.1) | 221 (43.5) |  |
|  | 5 and more | 59 (50.4) | 277 (53.3) |  | 60 (46.5) | 272 (53.5) |  |
| Young child present (< 2yrs) | | 12 (10.3) | 43 (8.3) | 0.61 | 18 (14.0) | 36 (7.1) | 0.02** |
| Kindergarten-age child present (2 - 5yrs) | | 58 (49.6) | 213 (41.0) | 0.11 | 58 (45.0) | 206 (40.6) | 0.42 |
| School-age child present (6 - 17yrs) | | 47 (40.2) | 246 (47.3) | 0.19 | 51 (39.5) | 242 (47.6) | 0.12 |
| ** Variable was included in Cox PH model | |  |  |  |  |  |  |
| ^ Missing value for one participant | |  |  |  |  |  |  |
